# Nystose treats intervertebral disc degeneration via the Nrf2 axis: a focus on oxidative stress and ferroptosis

**DOI:** 10.3389/fphar.2026.1842913

**Published:** 2026-07-15

**Authors:** Haifeng Mei, Xiuxiu Zheng, Dawei Han, Tao Xia, Yuhua Guo

**Affiliations:** 1 Department of Orthopaedics, Taizhou Hospital of Zhejiang Province Affiliated with Wenzhou Medical University, Linhai, Zhejiang, China; 2 Bone Development and Metabolism Research Center of Taizhou Hospital, Linhai, Zhejiang, China

**Keywords:** ferroptosis, intervertebral disc degeneration, Nrf2, nystose, oxidative stress

## Abstract

**Background:**

Intervertebral disc degeneration (IDD) is the primary aetiology of chronic lower back pain and is driven by factors such as oxidative stress and nucleus pulposus (NP) cell dysfunction. Nystose (Nys), a key active oligosaccharide derived from *Morinda officinalis* How., has shown potential in treating degenerative diseases; however, its specific effect and underlying mechanism of action in IDD remain largely unexplored.

**Purpose:**

This study aimed to investigate the therapeutic potential of Nys in IDD and elucidate whether its protective effects are mediated by the nuclear factor erythroid 2-related factor 2 (Nrf2)/haem oxygenase-1 (HO-1)/glutathione peroxidase 4 (GPX4) signalling axis.

**Methods:**

Network pharmacology was used to identify potential targets of Nys. *In vitro*, Nys–Nrf2 binding was predicted via molecular docking and thermal shift assays, and the effects of this interaction on ROS levels, ferroptosis, and extracellular matrix (ECM) metabolism were evaluated in oxidatively stressed NP cells. These effects were verified using Nrf2 siRNA. The *in vivo* efficacy of Nys was assessed in a lumbar spine instability (LSI) mouse model.

**Results:**

Network pharmacology identified Nrf2 as a core regulatory node. Nys suppressed ROS production and ferroptosis via iron metabolism regulation, which was driven by Nys binding to Nrf2 to promote its nuclear translocation. Nrf2 silencing abolished the ability of Nys to protect the ECM and exert antiferroptotic effects. *In vivo* testing confirmed that Nys shields the intervertebral disc from LSI-mediated damage through robust Nrf2 activation.

**Conclusion:**

Nystose alleviates IDD by activating the Nrf2/HO-1/GPX4 signalling axis, which in turn inhibits oxidative stress and ferroptosis to restore ECM homeostasis. Nys represents a promising therapeutic candidate for IDD intervention.

## 1 Introduction

Intervertebral disc degeneration (IDD) is a complex, chronic pathological process and represents the primary aetiology of clinical chronic lower back pain, imposing a heavy burden on middle-aged and elderly people worldwide (Knezevic et al., 2021). Clinically, IDD manifests as restricted spinal mobility, radicular pain, and significant impairment of lumbar function (Mohd Isa et al., 2022). Beyond mere physiological senescence, the onset of IDD is characterized by breakdown of the homeostatic balance of the nucleus pulposus (NP) cell microenvironment (Xia et al., 2024). Accumulating evidence suggests that in the presence of mechanical strain, metabolic dysregulation, and proinflammatory cytokines, the excessive accumulation of reactive oxygen species (ROS) in NP cells triggers severe oxidative stress (Wang et al., 2023). This oxidative burst triggers the upregulation of matrix metalloproteinases and a disintegrin and metalloproteinase with thrombospondin motifs, driving the massive breakdown of key ECM components such as type II collagen alpha 1 (COL2A1) and aggrecan (ACAN) (Mai et al., 2025; Chen X. et al., 2024). This imbalance in extracellular matrix (ECM) metabolism triggers NP dehydration and the subsequent loss of disc height (Hammoor et al., 2026). Consequently, reducing oxidative stress in NP cells represents a vital strategy for halting the progression of IDD. Current clinical management relies mainly on oral medications such as nonsteroidal anti-inflammatory drugs and muscle relaxants (Samanta et al., 2023). Although these conventional therapies offer transient palliative relief, their prolonged administration is frequently limited by varying degrees of gastrointestinal, cardiovascular, and hepatorenal toxicity (Albert et al., 2024; Vaudreuil et al., 2017). Given that functional impairment caused by IDD impose staggering economic and medical burdens on both families and society (Tolson et al., 2024), the use of highly safe natural compounds that target NP cell homeostasis has emerged as a promising avenue for the treatment of intervertebral disc degeneration.

Oxidative stress represents a state of imbalance between the generation of reactive oxygen species (ROS) and the activity of the antioxidant defence system (Yoshikawa and You, 2024). Oxidative stress is extensively involved in biological processes such as cellular damage, senescence, and metabolic dysregulation and thus dictates cell fate (Luo et al., 2025; Faraonio, 2022). In the hypoxic and ischaemic environment of the intervertebral disc, excessive ROS lead to increased levels of lipid peroxidation products (such as malondialdehyde (MDA)) and genomic instability through the direct oxidation of lipids, proteins, and DNA (Yang X. et al., 2023; Ma et al., 2023). This persistent oxidative damage serves not only as an initiating pathological factor in IDD but also as a driving force throughout the entire process. To counter the sustained injury caused by oxidative damage, cells possess a sophisticated defence system in which the nuclear factor erythroid 2-related factor 2 (Nrf2)/haem oxygenase-1 (HO-1) axis is recognized as the central regulatory hub (Zhang C. Y. et al., 2022; Xiang et al., 2022). Upon sensing oxidative stress signals, the nuclear factor Nrf2 undergoes rapid nuclear translocation, inducing the expression of the downstream antioxidant protein haem oxygenase-1 (HO-1) (Pan et al., 2023). The activation of HO-1 neutralizes free superoxide anions and modulates the intracellular redox potential, providing a metabolic buffer for nucleus pulposus (NP) cells (Yang G. et al., 2023). However, in severe IDD, this endogenous protective mechanism often fails because of overwhelming stress, leading to the collapse of the defensive barrier and subsequently triggering programmed cell death (Zhu et al., 2023; Li F. et al., 2024).

The collapse of this defence system precipitates ferroptosis, a modality of regulated cell death underpinned by the iron-dependent accumulation of lipid peroxides (Liu et al., 2022). In addition to the cytoprotection afforded by the Nrf2/HO-1 axis, elevated ROS levels increase the labile iron pool through the Fenton reaction, thereby activating a deleterious lipid peroxidation cascade (Nan et al., 2025). This imbalance in redox homeostasis culminates in the impairment of glutathione peroxidase 4 (GPX4), the primary enzyme that protects against ferroptotic insults (Zhang W. et al., 2024). The functional impairment of GPX4 deprives NP cells of their innate capacity to detoxify phospholipid hydroperoxides (Zhou et al., 2022). Consequently, the failure of the Nrf2-driven antioxidant response leaves cells vulnerable to the rapid production of lipid ROS, culminating in irreversible oxidative damage to membrane phosphoglycerides (Jomova et al., 2024). Within the microenvironment of the intervertebral disc, GPX4 depletion-mediated ferroptosis not only diminishes the viable cell population but also alters extracellular matrix (ECM) metabolism, eventually promoting the progression of intervertebral disc degeneration (IDD) towards an irreversible state (Zhang Y. et al., 2024).

Nystose (Nys; molecular weight: 666.58 g/mol) is a product extracted and refined from the dried roots of the dicotyledonous plant *Morinda officinalis* How. (Rubiaceae). Owing to its highly polar oligosaccharide structure, Nys has excellent water solubility, which facilitates its systemic absorption and bioavailability, thereby forming the basis for its pharmacological behaviour (Bali et al., 2015). *Morinda officinalis* How. possesses functions such as tonifying kidney yang, strengthening tendons and bones, dispelling wind and dampness, and improving immunity and is widely used in traditional medicine (Pawlus and Kinghorn, 2007). Modern research shows that *Morinda officinalis* oligosaccharides are the key active ingredients determining the therapeutic efficacy of *Morinda officinalis* How. (Li et al., 2021; Cao et al., 2024; Zhu et al., 2024), while Zhang et al. reported that Nys is a clear phytochemical marker for controlling the quality of medicinal *Morinda officinalis* oligosaccharides (Zhang Z. W. et al., 2022). Recent pharmacological studies have highlighted the diverse therapeutic properties of Nys, particularly its efficacy in managing musculoskeletal conditions, oxidative stress, and inflammation. For example, Nys has been shown to stimulate osteoblast differentiation and promote bone mineralization (Zhang et al., 2023). In the context of joint disease, it exerts significant anti-inflammatory effects by suppressing inflammasome activation during knee osteoarthritis (Wang et al., 2026). Beyond its benefits for skeletal health, Nys directly combats oxidative damage by upregulating the expression of proteins responsible for clearing superoxide radicals and maintaining cellular redox balance (Chen Y. et al., 2024). Together, these protective mechanisms strongly suggest that Nys has therapeutic potential for the treatment of IDD. This study aims to systematically analyse the effects of Nys on NP cell degeneration and IDD progression through network pharmacology studies and at the cellular, animal, and molecular levels, with a special focus on its ability to regulate oxidative stress and ferroptosis via the Nrf2/HO-1/GPX4 axis, providing a theoretical basis for the development of innovative IDD treatments.

## 2 Materials and methods

### 2.1 Prediction of the therapeutic targets of Nys in the treatment of IDD

To identify the therapeutic targets of Nys in the treatment of IDD, its 3D structure was retrieved from PubChem, screened via SuperPred (probability > 0), and standardized to filter for human targets using UniProt. IDD-related genes were retrieved from the Comparative Toxicogenomics Database (CTD) and GeneCards database (all relevance scores ≥ 5), and the intersecting genes served as the disease target set.

The overlapping targets between Nys and IDD were identified using a Venn diagram and subjected to protein‒protein interaction (PPI) analysis with STRING (v11.5). The resulting network was visualized in Cytoscape (v3.9.0), with nodes representing proteins and edges denoting interactions. To highlight regulatory hubs, the nodes were coloured according to degree values, with deeper red indicating more key proteins.

We utilized the Metascape database to perform comprehensive functional enrichment analyses. This involved evaluating WikiPathways, KEGG pathways, and Gene Ontology (GO) terms, including molecular function (MF), cellular component (CC), and biological process (BP) terms.

### 2.2 Reagents and antibodies

Nystose (Nys, ≥99% purity) and sulforaphane (SFN, ≥99% purity) were obtained from MedChemExpress (China). Tert-butyl hydroperoxide (TBHP) was obtained from Sigma‒Aldrich (United States; cat. no. 416665). Primary antibodies against matrix metalloproteinase 3 (MMP3; cat. no. ab52915) and HO-1 (cat. no. ab305290) were obtained from Abcam (United States), while an antibody against ACAN (cat. no. PA1-1746) was obtained from Thermo Fisher Scientific (UK). A range of antibodies, including those targeting COL2A1 (cat. no. ER1906-48), ADAM metallopeptidase with thrombospondin type 1 motif 4 (ADAMTS4; cat. no. ER1903-31), ferroportin-1 (SLC40A1; cat. no. ER 1916-80), the amino acid transport system Xc- (xCT; cat. no. HA600098), Nrf2 (cat. no. ER1706-41), GPX4 (cat. no. ET1706-45), NAD(P)H quinone dehydrogenase 1 (NQO1; cat. no. ER1802-85), superoxide dismutase 2 (SOD2; cat. no. ET1701-54), and β-actin (cat. no. HA610019), were purchased from Hangzhou HuaAn Biotechnology Co., Ltd., (China). Additionally, antibodies against ferritin light chain (FTL; cat. no. A1768) and transferrin receptor (TFR; cat. no. AF5343) were acquired from ABclonal (China) and Affinity (China), respectively. We purchased all secondary antibodies, which consisted of Alexa Fluor 488 (cat. no. HA1121), Alexa Fluor 594 (cat. no. HA1122), and a goat anti-rabbit antibody (cat. no. HA1001), from Hangzhou HuaAn Biotechnology.

### 2.3 Animal experiments

The experimental design was formally approved by the Ethics Committee of Taizhou Hospital (Approval code: tzyy2025209). We utilized 24 male C57BL/6 mice (8 weeks old, weighing 25 ± 3 g) obtained from Shanghai Slack Laboratory Animal Co. All the mice were given *ad libitum* access to sterilized water and a nutritionally balanced pellet diet. Throughout the study, we adhered strictly to the 3Rs principle (replacement, reduction, and refinement) to minimize animal distress.

To simulate IDD, we generated a lumbar spine instability (LSI) model. Following anaesthesia with isoflurane, the mice were placed in the prone position. A midline incision spanning L3 to L6 was made, and we performed targeted resection of the supraspinous and interspinous ligaments at the L4–L5 level. To further destabilize the segment, the bilateral facet joints were carefully excised. The sham group underwent an identical surgery (including skin incision and muscle separation) but without ligament or joint resection. Postoperative care included thermal regulation on a heating pad and monitoring for signs of neurological deficit. The mice were randomly divided into four cohorts (n = 6 per group): 1. The sham group, which received vehicle treatment postoperatively; 2. The LSI group, which underwent LSI surgery followed by vehicle treatment; 3. The LSI + Nys (10 mg/kg) group, which underwent LSI surgery and was treated with low-dose nystose; and 4. The LSI + Nys (20 mg/kg) group, which underwent LSI surgery and was treated with high-dose nystose. Nys was reconstituted in 0.9% saline containing 1% dimethyl sulfoxide (DMSO). Starting from the first day after surgery, treatments were administered via intraperitoneal injection twice weekly for a duration of 8 weeks.

### 2.4 Cell culture

Professor Di Chen from Rush University (United States) kindly donated the mouse NP cells employed in the current study. We cultured the NP cells in Dulbecco’s modified Eagle’s medium supplemented with 5% foetal bovine serum and 1% penicillin‒streptomycin. To ensure optimal growth, we replaced the culture medium every 48 h while keeping the cells in a 37 °C humidified environment with 5% CO_2_. Upon reaching approximately 90% confluence, an *in vitro* model of oxidative damage was established by exposing the cells to TBHP (0 or 100 μM) for 4 h (Ma et al., 2025). Subsequently, the cells were treated with either Nys or a positive control SFN (10 μM, a classical Nrf2 agonist (Lu et al., 2023)) to evaluate comparative efficacy.

### 2.5 Cell viability assay

We used a Cell Counting Kit-8 (CCK-8) to systematically measure how Nys exposure altered NP cell proliferation. First, NP cells were seeded into 96-well plates at a density of 5 × 10^3^ cells/well and cultured overnight to ensure stable adherence. The cells were then exposed to various concentrations of Nys (0, 3.125, 6.25, 12.5, 25, 50, 100, 200, 400, and 800 μM) for either 24 or 48 h. Afterwards, 10 μL of CCK-8 reagent was added to each well for exactly 1 h at 37 °C. The optical density (OD) was subsequently measured at a wavelength of 450 nm using a Multiskan FC microplate luminometer (Thermo Fisher Scientific, United States). Finally, the cell survival rates were calculated and normalized to those of the untreated control group.

### 2.6 High-density cell culture

To evaluate the impact of Nys on the stability of the ECM, a high-density cell culture was established. NP cells were harvested and resuspended in complete medium at a density of 1 × 10^7^ cells/mL. A 10 μL droplet of this cell suspension was precisely placed in the centre of each well of a 24-well plate. Following a 4-h preadherence period at 37 °C, 500 μL of complete medium was gently added to each well. After a 24-h stabilization phase, the micromasses were challenged with TBHP (0 or 100 μM) and treated with varying concentrations of Nys (0, 25, or 50 μM) for 7 days. At the experimental endpoint, the cultures were fixed with 4% paraformaldehyde (PFA) and subjected to toluidine blue staining to visualize proteoglycans. Stained micromasses were subsequently imaged using an EPSON V600 photo scanner (Japan) for qualitative and quantitative analysis of ECM integrity.

### 2.7 Quantitative real-time PCR (RT‒qPCR)

To obtain total RNA from the NP cells, we utilized the RN28-EASYspin Plus Tissue/Cell Rapid Extraction Kit (Aidlab Biotechnology, China) with strict adherence to the manufacturer’s guidelines. Following extraction, both the concentration and the quality of the recovered RNA were quantified via a NanoDrop 2000 spectrophotometer (Thermo Fisher Scientific, United States). First-strand cDNA synthesis was accomplished via a Cwbiotech HiFiScript kit, with all reaction steps performed according to the manufacturer’s recommended guidelines. Quantitative PCR was conducted on an ABI 7300 Plus Real-Time PCR System (Applied Biosystems, United States) utilizing SYBR Green Premix Ex Taq (Takara, Japan). The thermal cycling conditions were as follows: initial denaturation at 95 °C for 30 s, followed by 40 cycles of 95 °C for 5 s and 60 °C for 30 s. The relative mRNA expression levels of target genes were calculated using the 2^−ΔΔCt^ method and normalized to those of the endogenous control, β-actin. The primer pairs utilized are listed in Table 1.

**TABLE 1:** Sequences of the primers used for real-time PCR.

Gene	Primer sequence (5′–3′)	
HO-1	Forward reverse	CCCAAAACTGGCCTGTAAAA CGTGGTCAGTCAACATGGAT
SOD2	Forward reverse	GAGGCTATCAAGCGTGACTTTG GCAATGGGTCCTGATTAGAGC
NQO1	Forward reverse	ATCACCAGGTCTGCAGCTTC GCCATGAAGGAGGCTGCTGT
COL2A1	Forward reverse	TGA​CCT​GAC​GCC​CAT​TCA​TC TTT​CCT​GTC​TCT​GCC​TTG​ACC​C
ACAN	Forward reverse	GCAGCACAGACACTTCAGGA CCCACTTTCTACAGGCAAGC
ADAMTS4	Forward reverse	CGC​TGA​GTA​GAT​TCG​TGG​AGA​C AGT​TGA​CAG​GGT​TTC​GGA​TGC
MMP3	Forward reverse	CCC​TGA​TGT​CCT​CGT​GGT​A GGT​CCT​GAG​AGA​TTT​TCG​CC
β-Actin	Forward reverse	AGCCATGTACGTAGCCATCC ACCCTCATAGATGGGCACAG

### 2.8 Western blotting (WB)

Cellular proteins were extracted using RIPA buffer (Beyotime, China) supplemented with 1% PMSF and 2% phosphatase inhibitors. After being subjected to ice-cold lysis (30 min) and centrifugation (12,000 rpm, 15 min, 4 °C), the supernatants (AMEKO, China), whose protein concentrations had been quantified with a BCA kit, were resolved via SDS‒PAGE (10 μL/lane) and blotted onto Millipore PVDF membranes. The blots were blocked in 5% skim milk for 2 h, probed with primary antibodies overnight at 4 °C, and subsequently washed three times in TBST. Following 1 h of incubation with HRP-linked secondary antibodies at ambient temperature, the immunoreactive bands were visualized with ECL reagents (Millipore), captured on an ImageQuant LAS-500 system (GE Life Sciences), and densitometrically analysed with ImageJ.

### 2.9 Immunofluorescence (IF) staining

The distribution and expression of target proteins within NP cells were visualized via IF staining. First, treated cells were fixed with 4% PFA for 15 min and subsequently permeabilized using 0.1% Triton X-100 for 10 min. The samples were blocked in 5% BSA (1 h, ambient temperature) and probed with primary antibodies overnight at 4 °C in the dark. After being washed with PBS, the cells were incubated with Alexa Fluor 488/594-conjugated secondary antibodies for 60 min and with DAPI nuclear stain for 5 min. A confocal microscope was used for image acquisition, followed by ImageJ-based quantification of the fluorescence intensity.

### 2.10 Flow cytometry (FC)

We measured intracellular ROS levels using a specific assay kit (Elabscience, cat. no. E-BC-K138-F). Cells were rinsed with PBS and incubated for 30 min in the dark at 37 °C with 10 μM DCFH-DA probe. After staining, the samples were centrifuged (1,000 × *g*, 5 min) and cleared of residual dye by washing with serum-free medium. Finally, we recorded the fluorescence intensity for 3,000 events per group with a Beckman Coulter cytometer and quantitatively analysed the data via CytExpert.

### 2.11 Enzyme-linked immunosorbent assay (ELISA)

The intracellular levels of MDA and glutathione (GSH) were quantified using specific ELISA kits (Elabscience Biotechnology, China; cat. nos. E-EL-0060 and E-EL-0026) according to the manufacturer’s instructions. Briefly, NP cells were harvested and lysed through repeated freeze‒thaw cycles, followed by centrifugation at 1,500 × *g* for 10 min at 4 °C to collect the supernatant. For both assays, 50 μL of standard or sample was added to micro-ELISA plates to compete with precoated antigens for binding sites on the biotinylated detection antibodies. After sequential incubation with HRP-conjugated antibodies and 3,3′,5,5′-tetramethylbenzidine substrate, the reaction was terminated, and the optical density was measured at 450 nm. The concentrations were calculated using standard curves and normalized to the total protein concentration as determined by the BCA assay.

### 2.12 Ferrous iron assay

Intracellular ferrous iron (Fe^2+^) levels were quantified utilizing a Cell Fe^2+^ Fluorometric Assay Kit (Elabscience Biotechnology, China; cat. no. E-BC-F101). In brief, NP cells were seeded into 96-well black microplates and allowed to adhere overnight. Following the experimental treatments, the cells were incubated with 5 μM probe working solution at 37 °C in the dark for 60 min. The fluorescence intensity was quantified via a microplate reader (excitation/emission wavelengths = 542/575 nm). The final Fe^2+^ concentrations were determined on the basis of a standard curve and normalized for comparative analysis.

### 2.13 Molecular docking simulation

To explore binding interactions between nystose and target proteins, molecular docking simulations were conducted. The chemical structure of the ligand was retrieved from the PubChem database and subsequently converted from SDF to PDB format utilizing Open Babel (version 2.3.2). The three-dimensional coordinates for the target protein were sourced from the RCSB Protein Data Bank. Preprocessing of the protein structure, including the removal of crystal water molecules and original ligands, was performed using PyMOL (version 2.3.4). Afterwards, AutoDockTools was employed to add polar hydrogen atoms and assign Gasteiger charges to the receptor proteins. Both the prepared proteins and ligand molecules were saved in PDBQT format for compatibility. Molecular docking simulations were executed using AutoDock Vina (version 1.1.2), and the resulting binding poses were studied for noncovalent interactions using the Protein‒Ligand Interaction Profiler. The final docking conformations and interaction residues were visualized and rendered using PyMOL.

### 2.14 Cellular thermal shift assay (CETSA)

Direct interactions between nystose and its candidate target proteins were evaluated via CETSAs. Briefly, 1 × 10^7^ NP cells were collected and resuspended in ice-cold PBS supplemented with 1% protease inhibitor cocktail. The suspension underwent three successive freeze‒thaw cycles in liquid nitrogen. The raw lysates were subsequently clarified by centrifugation at 12,000 × *g* for 15 min at 4 °C to isolate the soluble protein fraction. The resulting supernatants were separated into two equivalent cohorts and treated with either 50 μM Nys or the vehicle control DMSO for 30 min at 25 °C. Following incubation, samples from each group were further divided into 7 equal aliquots and subjected to a thermal gradient (ranging from 40 °C to 70 °C at 5 °C intervals) for 5 min per sample. After heating, the samples were again centrifuged at 12,000 × *g* for 15 min at 4 °C to precipitate any thermally denatured proteins. The remaining soluble proteins in the supernatant were recovered and denatured in loading buffer, and their expression was quantified via Western blotting to determine the changes in melting temperature.

### 2.15 Transmission electron microscopy (TEM)

To evaluate ferroptotic changes at the ultrastructural level, transmission electron microscopy was performed. Following the appropriate treatments, nucleus pulposus (NP) cells were harvested, centrifuged to obtain pellets, and immediately fixed with 2.5% glutaraldehyde in 0.1 M phosphate buffer (pH 7.4) at 4 °C overnight. The samples were subsequently washed three times with phosphate buffer and postfixed with 1% osmium tetroxide (OsO4) for 2 h at room temperature. After extensive washing, the cell pellets were dehydrated through a graded series of ethanol solutions (50%, 70%, 80%, 90%, 95%, and 100%) and embedded in epoxy resin. Ultrathin sections (approximately 70–80 nm thick) were cut using an ultramicrotome, collected on copper grids, and double-stained with 2% uranyl acetate and lead citrate. Mitochondrial morphology and cellular ultrastructure were ultimately observed and imaged using a transmission electron microscope.

### 2.16 Small interfering RNA (siRNA) transfection

We achieved targeted Nrf2 depletion by transfecting cultured NP cells with a 50 nM siRNA construct (Suzhou Ribo Life Science; cat. no. siG150114100151-1-5). The siRNA transfection complexes were prepared following the manufacturer’s instructions and added dropwise to antibiotic-free culture medium. The final concentration of siRNA in each well was 50 nM. Cells were incubated for 24 h at 37 °C to allow for gene silencing before subsequent treatments.

### 2.17 Histopathological analysis of spinal tissues

After 8 weeks of treatment, the model mice were euthanized by an overdose of isoflurane. L4–L5 spinal tissues were harvested and fixed in 4% paraformaldehyde for 48 h and then decalcified in a 10% ethylenediaminetetraacetic acid solution. After confirming decalcification, the samples were dehydrated in ethanol, cleared in xylene, embedded in paraffin, and sectioned. The sections were stained with haematoxylin‒eosin (HE) and safranin O-fast green (SO) to evaluate the morphological integrity of the NP and the tissue structure of the annulus fibrosus (AF). A new scoring system based on mouse intervertebral disc histomorphology established by Vivian Tam et al. was used to assess the cell density of the NP and the integrity of the AF. The scores were evaluated by two independent observers in a blinded manner.

### 2.18 Immunohistochemical (IHC) staining

Paraffin sections were deparaffinized and rehydrated with ethanol. Antigen retrieval was performed using sodium citrate, and 3% hydrogen peroxide was used to remove endogenous peroxidase activity. After blocking with 5% bovine serum albumin, the sections were incubated with primary antibody against the target protein overnight at 4 °C. We subsequently applied secondary antibodies for 60 min at room temperature, DAB was used for colorimetric detection, and the sections were counterstained with haematoxylin. The resin-mounted slides were ultimately evaluated microscopically.

### 2.19 Statistical analysis

Data from at least five independent replicates are expressed as the mean ± SD. After the normality of the data was evaluated by the Shapiro‒Wilk test, significance (P < 0.05) was determined using Student's t tests (two groups) or one-way analysis of variance (multiple groups) via GraphPad Prism 9.0. Nonparametric equivalents were used for heteroscedastic or nonnormally distributed datasets.

## 3 Results

### 3.1 Network pharmacology analysis of nys and IDD

**FIGURE 1:**
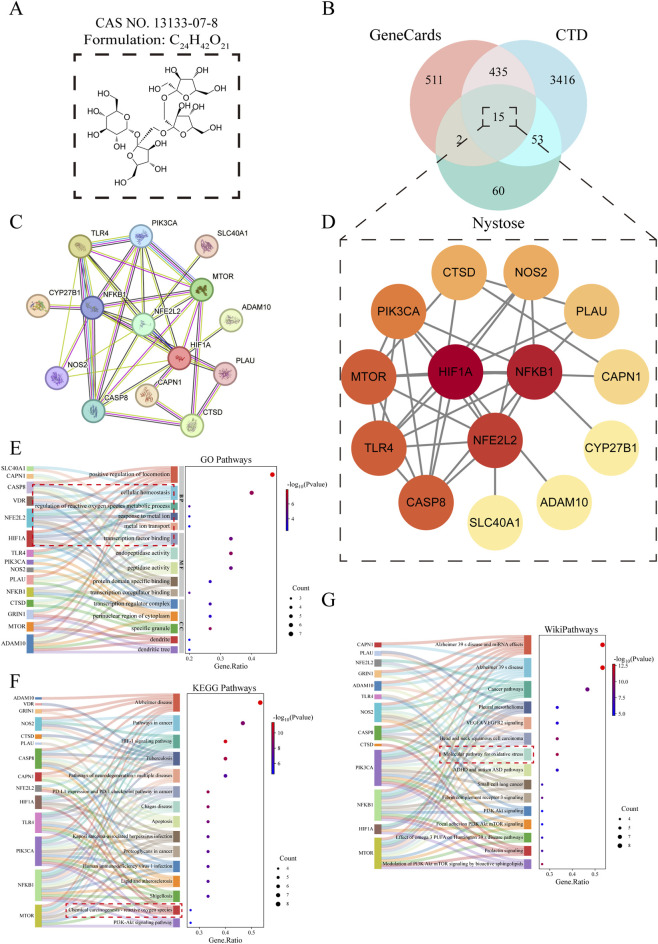
Network pharmacology analysis of nystose (Nys) and intervertebral disc degeneration (IDD). **(A)** Chemical structure and CAS number of Nys. **(B)** Venn diagram showing overlapping genes between Nys targets and IDD-related genes. **(C,D)** Protein‒protein interaction (PPI) network and visualization of the overlapping genes. **(E)** Results of the GO enrichment analysis of the overlapping genes. **(F)** The top 15 enriched KEGG pathways associated with the overlapping targets of Nys and IDD. **(G)** The top 15 enriched Wikipathways for the overlapping genes.

To explore the pharmacological basis of the effect of Nys in the treatment of IDD, we first identified the potential targets of Nys through network pharmacology analysis. The chemical structure of Nys is shown in Figure 1A. Target prediction database analysis revealed 130 target proteins related to Nys, while 450 potential therapeutic targets for IDD were predicted from disease target databases. There were 15 overlapping targets identified as potential targets of Nys in the treatment of IDD (Figure 1B). The PPI network constructed on the basis of these overlapping targets contained 14 nodes and 35 edges (Figures 1C,D). The top 5 ranked targets were HIF1A, NFKB1, NFE2L2, TLR4, and MTOR (Table 2). We subsequently performed functional enrichment analysis of these overlapping targets. GO enrichment analysis revealed that the relevant targets play important roles in maintaining cellular homeostasis and regulating oxidative stress, transcription factor binding, and metal ion regulation (Figure 1E). According to the top 15 significantly enriched KEGG pathways and WikiPathways, Nys may exert therapeutic effects on IDD by regulating oxidative stress-related functions (Figures 1F,G). Notably, the iron metabolism-related gene SLC40A1 was also enriched. Although the PPI network revealed several core targets, such as HIF1A and NFKB1, the results of the GO and KEGG analyses strongly indicated the involvement of oxidative stress and metal ion regulation. Among the top-ranked targets, NFE2L2 (Nrf2) is well documented as a central transcription factor that regulates both antioxidant responses and iron homeostasis (Nan et al., 2025; Xiang et al., 2024). Since oxidative damage and ferroptosis are critical drivers of IDD (Fan et al., 2023), Nrf2 has emerged as a logical molecular hub connecting the predicted pharmacological effects of Nys with IDD pathology. On the basis of this rationale, we focused our subsequent *in vitro* and *in vivo* experiments on the Nrf2 signalling pathway.

**TABLE 2:** Top 5 targets in the PPI network ranked by degree score.

No.	Name	Degree	Protein name
1	HIF1A	10	Hypoxia inducible factor 1 subunit alpha
2	NFKB1	9	Nuclear factor kappa B subunit 1
3	NFE2L2	8	Nuclear factor erythroid 2-related factor 2
4	TLR4	7	Toll like receptor 4
5	MTOR	7	Mammalian target of rapamycin

### 3.2 Nys alleviates TBHP-Induced oxidative stress

**FIGURE 2:**
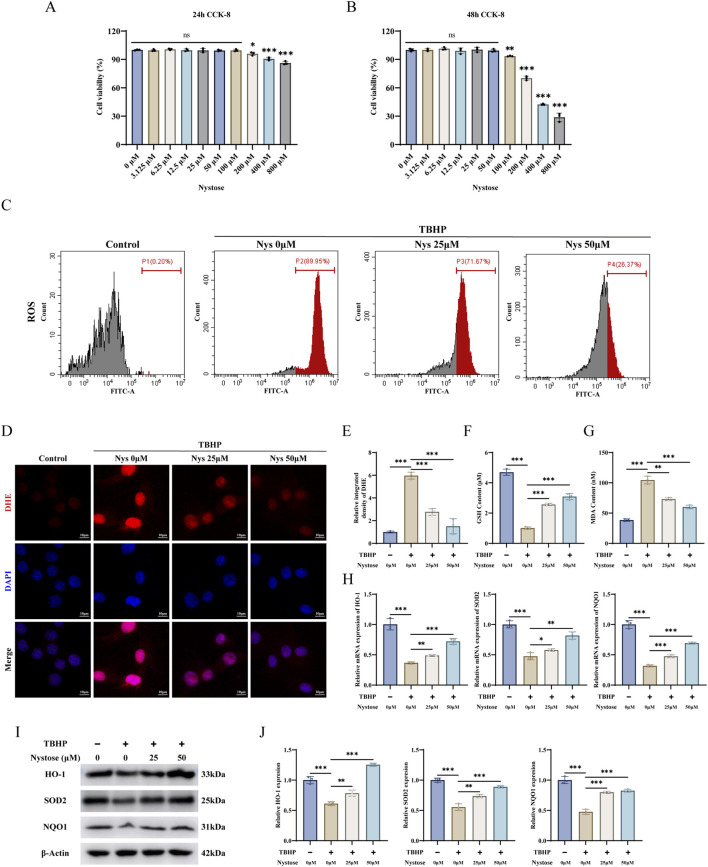
Nystose (Nys) alleviates tert-butyl hydroperoxide-induced oxidative stress. **(A,B)** Results of a cell counting kit-8 (CCK-8) assay involving nucleus pulposus (NP) cells after 24 or 48 h of Nys treatment (n = 3). **(C)** Flow cytometry analysis of ROS levels in NP cells under different treatments (n = 3). **(D,E)** Immunofluorescence staining showing DHE expression levels in NP cells subjected to different treatments, with relative expression levels quantified using ImageJ (scale bar = 10 μm; n = 3). **(F,G)** Changes in MDA and GSH contents in NP cells after 24 h of different treatments (n = 3). **(H)** Relative mRNA expression of HO-1, SOD2, and NQO1 in NP cells after different treatments (n = 3). **(I,J)** Western blot analysis of HO-1, SOD2, and NQO1 expression in NP cells after different treatments; relative expression levels were quantified using ImageJ (n = 3). Statistical significance was determined using Student’s t-tests for two groups or one-way ANOVA for multiple comparisons. The data are expressed as the mean ± standard deviation. *p < 0.05, **p < 0.01, ***p < 0.001.

To clarify the effect of Nys on the viability of NP cells, we assessed the activity of NP cells under stimulation with different concentrations of Nys through CCK-8 assays. After 24 or 48 h of stimulation, NP cell viability was not inhibited at a Nys concentration of 50 μM or less (Figures 2A,B). Moreover, we calculated the IC_50_ of Nys in NP cells at 48 h to be 237.4 μM. In subsequent *in vitro* experiments, 25 and 50 μM Nys was employed to ensure peak biological activity at nontoxic concentrations. We used TBHP to establish an NP cell oxidative stress model to simulate the *in vivo* microenvironment of the nucleus pulposus (Ma et al., 2025). To evaluate the antioxidant capacity of Nys, we used the probe DCFH-DA to measure intracellular ROS levels via flow cytometry. The results indicated that TBHP significantly increased the content of intracellular ROS, but the ROS level in NP cells significantly decreased after the addition of Nys (Figure 2C). Moreover, immunofluorescence staining performed using the probe DHE also proved that Nys could significantly reduce ROS levels in NP cells under oxidative stress (Figures 2D,E). We subsequently found that Nys treatment significantly increased the GSH content and reduced MDA production in NP cells subjected to oxidative stress (Figures 2F,G). GSH is among the most important antioxidants in the human body, whereas MDA is among the most important products of membrane lipid peroxidation. Finally, through RT‒qPCR and WB, we confirmed that Nys promoted the gene transcription and protein expression of the antioxidant factors HO-1, SOD2, and NQO1 in NP cells under oxidative stress (Figures 2H–J). These results indicate that Nys can alleviate TBHP-induced oxidative stress in NP cells.

### 3.3 Nys attenuates oxidative stress-induced NP cell degeneration

**FIGURE 3:**
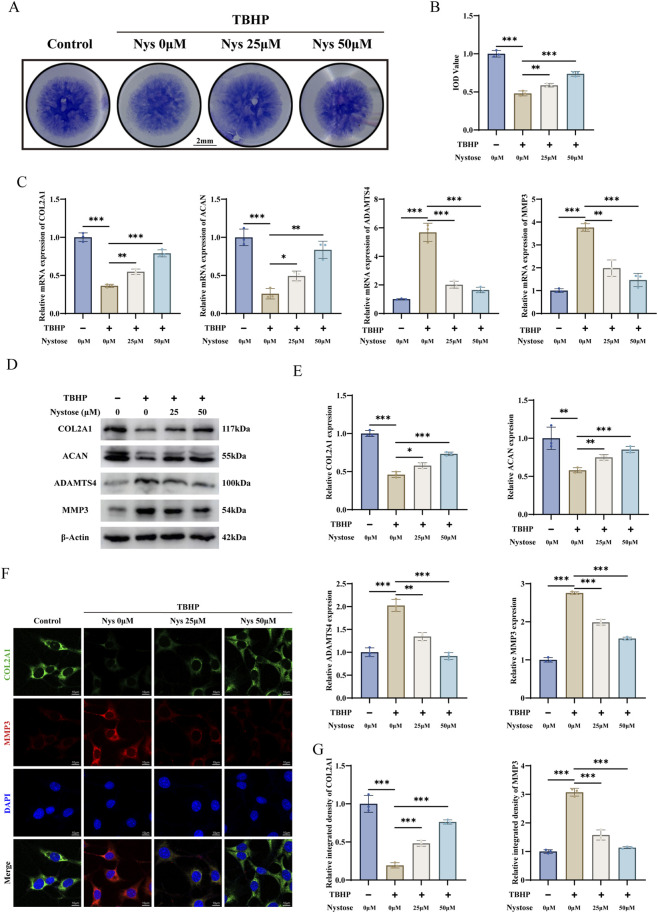
Nystose (Nys) attenuates oxidative stress-induced nucleus pulposus (NP) cell degeneration. **(A,B)** Representative images of toluidine blue-stained NP cells cultured at high density for 1 week (scale bar = 2 mm). The integrated optical density (IOD) was quantified using ImageJ software (n = 3). **(C)** Relative mRNA expression of COL2A1, ACAN, ADAMTS4, and MMP3 in NP cells after different treatments (n = 3). **(D,E)** Western blot analysis of COL2A1, ACAN, ADAMTS4, and MMP3 expression in NP cells after different treatments, with relative expression levels quantified using ImageJ (n = 3). **(F,G)** Immunofluorescence staining of COL2A1 and MMP3 expression in NP cells under different treatments, with the relative fluorescence intensity quantified using ImageJ (scale bar = 10 μm; n = 3). The data are expressed as the mean ± standard deviation. Statistical significance was determined using Student’s t-tests for two groups or one-way ANOVA for multiple comparisons. *p < 0.05, **p < 0.01, ***p < 0.001.

ECM components expressed and secreted by NP cells are key to maintaining the biological function of the intervertebral disc (Hwang et al., 2014). We reconstructed the rich extracellular matrix microenvironment of NP cells through high-density culture and found that Nys could alleviate the decrease in the levels ECM components caused by TBHP (Figures 3A,B). Moreover, the results of PCR and WB revealed that TBHP-induced oxidative stress led to decreases in the transcriptional activity and protein expression of the extracellular matrix components COL2A1 and ACAN, whereas the transcriptional activity and protein expression of MMP3 and ADAMTS4, key metabolic enzymes for ECM degradation, significantly increased. These findings indicate that the synthesis of ECM components by NP cells decreased while their degradation increased. However, Nys reversed this phenomenon, promoting the synthesis of ECM components and inhibiting their degradation under oxidative stress conditions (Figures 3C–E). We subsequently observed the same phenomenon through immunofluorescence, with Nys promoting the expression of COL2A1 and inhibiting the expression of ADAMTS4 under oxidative stress (Figures 3F,G). These results indicate that Nys can alleviate NP cell degeneration, thereby regulating the balance of ECM synthesis and catabolism in NP cells under oxidative stress.

### 3.4 Nys attenuates oxidative stress-induced ferroptosis

**FIGURE 4:**
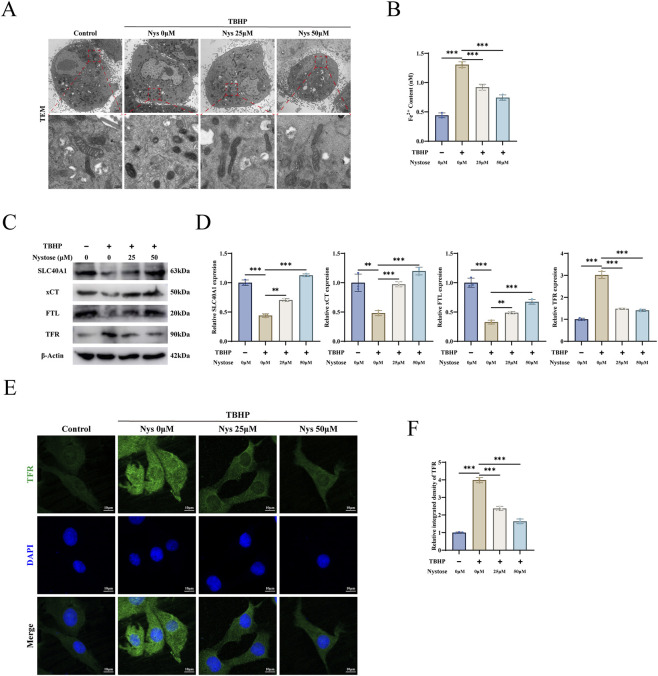
Nystose (Nys) attenuates oxidative stress-induced ferroptosis. **(A)** Representative transmission electron microscopy (TEM) images showing ferroptotic ultrastructural changes in nucleus pulposus (NP) cells under different treatments (scale bar = 1 μm/200 nm; n = 3). **(B)** Changes in the ferrous iron (Fe2+) content in NP cells after 24 h of different treatments (n = 3). **(C,D)** Western blot analysis of SLC40A1, xCT, FTL, and TFR expression in nucleus pulposus (NP) cells after different treatments, with relative expression levels quantified using ImageJ (n = 3). **(E,F)** Immunofluorescence staining of TFR expression in NP cells under different treatments, with the relative fluorescence intensity quantified using ImageJ (scale bar = 10 μm; n = 3). The data are expressed as the mean ± standard deviation. Statistical significance was determined using Student’s t-tests for two groups or one-way ANOVA for multiple comparisons. **p < 0.01, ***p < 0.001.

Ferroptosis is among the forms of cell death that are most directly driven by oxidative stress (Yu et al., 2021), and previous network pharmacology analyses suggested that Nys might be associated with ion transport and metabolism. To confirm ultrastructurally that ferroptosis occurred, TEM was performed. After TBHP exposure, NP cells displayed classic signs of ferroptosis, such as obvious mitochondrial shrinkage, membrane condensation, and a loss of cristae. However, the addition of Nys successfully reversed these structural defects and maintained normal mitochondrial morphology (Figure 4A). Furthermore, we measured the levels of intracellular ferrous ions. TBHP-induced oxidative stress led to a sharp increase in the levels of intracellular free ferrous ions, whereas Nys significantly alleviated oxidative stress-induced intracellular iron overload (Figure 4B). We subsequently analysed the expression of ferroptosis-related proteins through WB. The results revealed that TBHP-induced oxidative stress significantly downregulated the expression of the iron export protein SLC40A1, FTL, and the cystine transporter xCT while upregulating the expression of the iron intake protein TFR. These findings indicate that intracellular iron uptake and release are promoted, whereas iron storage and efflux are inhibited, ultimately leading to intracellular iron overload and increasing the likelihood of cellular lipid peroxidation. However, Nys treatment effectively upregulated SLC40A1, xCT, and FTL expression and downregulated TFR expression, thereby restoring intracellular iron homeostasis (Figures 4C,D). Immunofluorescence staining further verified this phenomenon, as the increase in TFR fluorescence intensity caused by TBHP was reversed by Nys (Figures 4E,F). In summary, Nys antagonizes oxidative stress-induced ferroptosis by preventing mitochondrial ultrastructural damage, regulating ferroptosis-related proteins and restoring iron homeostasis.

### 3.5 Nys regulates Nrf2 transcription and increases the expression of the key ferroptosis-regulating protein GPX4

**FIGURE 5:**
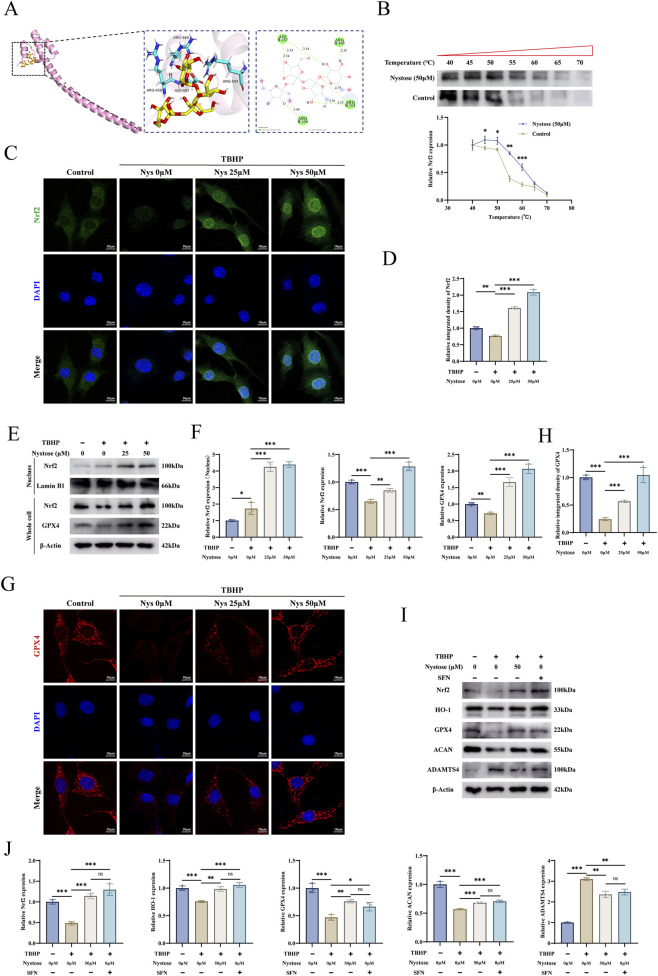
Nystose (Nys) regulates Nrf2 transcription and increases the expression of the key ferroptosis regulatory protein GPX4. **(A)** Three-dimensional (3D) and two-dimensional (2D) structural images showing the interaction between Nys and Nrf2. **(B)** The protein expression of Nrf2 was determined by CETSA, and its relative expression levels were quantified using ImageJ (n = 3). **(C,D)** Immunofluorescence staining of Nrf2 expression in nucleus pulposus (NP) cells under different treatments, with the relative fluorescence intensity quantified using ImageJ (scale bar = 10 μm; n = 3). **(E,F)** Western blot analysis of Nrf2 and GPX4 expression in NP cells after different treatments, with relative expression levels quantified using ImageJ (n = 3). **(G,H)** Immunofluorescence staining of GPX4 expression in NP cells under different treatments, with the relative fluorescence intensity quantified using ImageJ (scale bar = 10 μm; n = 3). **(I,J)** Western blot analysis evaluating the effects of Nys and the positive control sulforaphane (SFN) on the protein expression of Nrf2, HO-1, GPX4, COL2A1, and ADAMTS4 under TBHP-induced oxidative stress conditions, with relative expression levels quantified using ImageJ (n = 3). The data are expressed as the mean ± standard deviation. Statistical significance was determined using Student’s t-tests for two groups or one-way ANOVA for multiple comparisons. **p < 0.01, ***p < 0.001.

To explore the mechanism through which Nys regulates iron metabolism and the antioxidant defence system, we first predicted the interaction between Nys and the transcription factor Nrf2 through molecular docking simulations. The results indicated that Nys could stably bind the Kelch domain of Nrf2, with a binding energy of −4.9 kcal/mol (Figure 5A). 3D and 2D docking diagrams revealed that Nys primarily interacts with Nrf2 through hydrogen bonding with key amino acid residues, including ARG-456, ASP-457, ARG-460, and ARG-504, indicating a putative binding interaction between the two. The binding of Nys to Nrf2 was subsequently further assessed using CETSAs. Compared with the controls, Nys significantly increased Nrf2 thermal stability across the 40 °C–70 °C gradient, as indicated by a rightward-shifted degradation curve (Figure 5B).

As Nrf2 is a core transcription factor involved in the antioxidant response, its activation depends on its nuclear translocation (He et al., 2020). Immunofluorescence staining revealed that under resting conditions, Nrf2 was localized mainly in the cytoplasm. TBHP-induced oxidative stress led to the significant downregulation of Nrf2 expression, whereas Nys treatment markedly increased the nuclear translocation of Nrf2, as indicated by a significant increase in the intranuclear fluorescence intensity (Figures 5C,D). Nrf2 activation can initiate the transcription of downstream antioxidant genes. As a core executor of ferroptosis, GPX4 is among the important target genes of Nrf2 (Kajarabille and Latunde-Dada, 2019). Western blotting results confirmed that compared with those in the groups treated with TBHP alone, the intranuclear Nrf2 protein level in the Nys-treated group was significantly increased, accompanied by increases in Nrf2 and GPX4 levels in total cell lysates (Figures 5E,F). These findings suggest that Nys not only promotes Nrf2 nuclear translocation but also increases Nrf2 and GPX4 protein expression. Immunofluorescence staining revealed that the fluorescence intensity of GPX4 decreased in the presence of TBHP but significantly increased after Nys treatment (Figures 5G,H). To further verify the therapeutic potential of Nys, we used the classic Nrf2 agonist SFN as a positive control (Houghton et al., 2016). Western blot analysis revealed that under TBHP-induced oxidative stress conditions, both Nys and SFN robustly upregulated the protein expression of Nrf2 and its downstream targets, HO-1 and GPX4. Furthermore, regarding the maintenance of ECM homeostasis, Nys effectively restored the synthesis of COL2A1 and significantly suppressed the expression of the degradative enzyme ADAMTS4, demonstrating an overall protective effect that is highly consistent with that of the positive control SFN (Figures 5I,J). Collectively, these findings suggest that Nys positively regulates Nrf2, facilitating its nuclear translocation and the subsequent activation of GPX4 to strengthen the cellular antioxidant defence system, thereby protecting NP cells against ferroptosis with an efficacy fully comparable to that of established Nrf2 agonists.

### 3.6 The antioxidant and antiferroptotic effects of nys depend on nrf2/HO-1/GPX4 signalling

**FIGURE 6:**
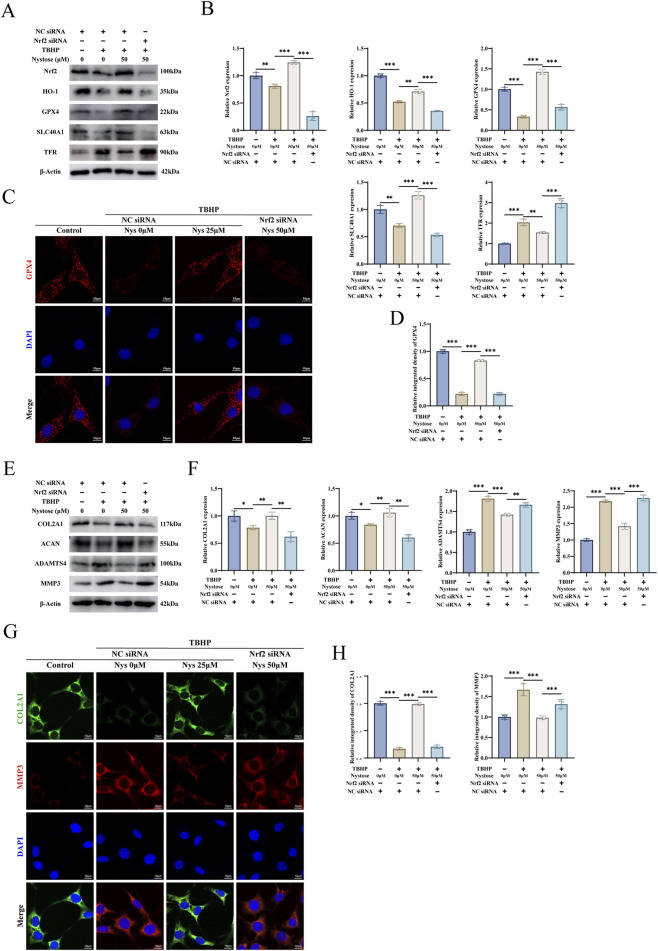
Nystose (Nys) regulates oxidative stress and ferroptosis in nucleus pulposus (NP) cells via the Nrf2/HO-1/GPX4 axis. **(A,B)** Western blot analysis of Nrf2, HO-1, GPX4, SLC40A1, and TFR expression in NP cells following Nrf2 silencing, with relative expression levels quantified using ImageJ (n = 3). **(C,D)** Immunofluorescence staining of GPX4 expression in NP cells following Nrf2 silencing, with the relative fluorescence intensity quantified using ImageJ (scale bar = 10 μm; n = 3). **(E,F)** Western blot analysis of COL2A1, ACAN, ADAMTS4, and MMP3 expression in NP cells following Nrf2 silencing, with relative expression levels quantified using ImageJ (n = 3). **(G,H)** Immunofluorescence staining of COL2A1 and MMP3 expression in NP cells following Nrf2 silencing, with the relative fluorescence intensity quantified using ImageJ (scale bar = 10 μm; n = 3). The data are expressed as the mean ± standard deviation. Statistical significance was determined using Student’s t-tests for two groups or one-way ANOVA for multiple comparisons. *p < 0.05, **p < 0.01, ***p < 0.001.

To verify the pivotal role of Nrf2 in the regulation of oxidative stress and ferroptosis in NP cells by Nys, we used siRNA to silence Nrf2 expression and evaluated its effect on the protective effects of Nys. First, Western blotting revealed that Nrf2 siRNA transfection significantly silenced Nrf2 expression. Consistent with previous results, TBHP treatment significantly inhibited the expression of Nrf2 and its downstream target genes HO-1 and GPX4 while downregulating the expression of the iron export protein SLC40A1 and upregulating the expression of the iron intake protein TFR. Nys treatment significantly activated the Nrf2/HO-1/GPX4 pathway, thereby reversing the TBHP-induced alterations in SLC40A1 and TFR expression. However, when Nrf2 was silenced by siRNA, the regulatory effects of Nys on these proteins were significantly blocked, as indicated by the failed recovery of HO-1 and GPX4 expression, with SLC40A1 remaining at low levels and TFR maintaining high expression (Figures 6A,B). Immunofluorescence staining of GPX4 further verified these results. Nrf2 siRNA transfection significantly abolished the upregulatory effect of Nys on GPX4 expression, confirming the important role of Nrf2 in the regulation of GPX4 expression by Nys (Figures 6C,D).

Furthermore, we investigated whether Nrf2 is also essential for Nys to maintain ECM homeostasis under oxidative stress. Western blotting revealed that while Nys treatment effectively restored the synthesis of ECM components (COL2A1 and ACAN) and inhibited degradative enzymes (ADAMTS4 and MMP3), these protective effects were significantly abrogated following Nrf2 silencing (Figures 6E,F). This shift from protection to degradation was further confirmed by double immunofluorescence staining, which showed that the Nys-mediated restoration of COL2A1 expression and suppression of MMP3 expression were largely reversed by Nrf2 interference, resulting in low COL2A1 and high MMP3 expression (Figures 6G,H).

These results indicate that Nrf2 is the core upstream mediator of Nys activity. Nys acts on the core transcription factor Nrf2 to drive the expression of the downstream factors HO-1 and GPX4, thereby synergistically regulating iron metabolism-related proteins and ECM metabolism and ultimately achieving a protective effect on NP cells.

### 3.7 Nys alleviates nucleus pulposus degeneration and delays IDD progression in vivo

**FIGURE 7:**
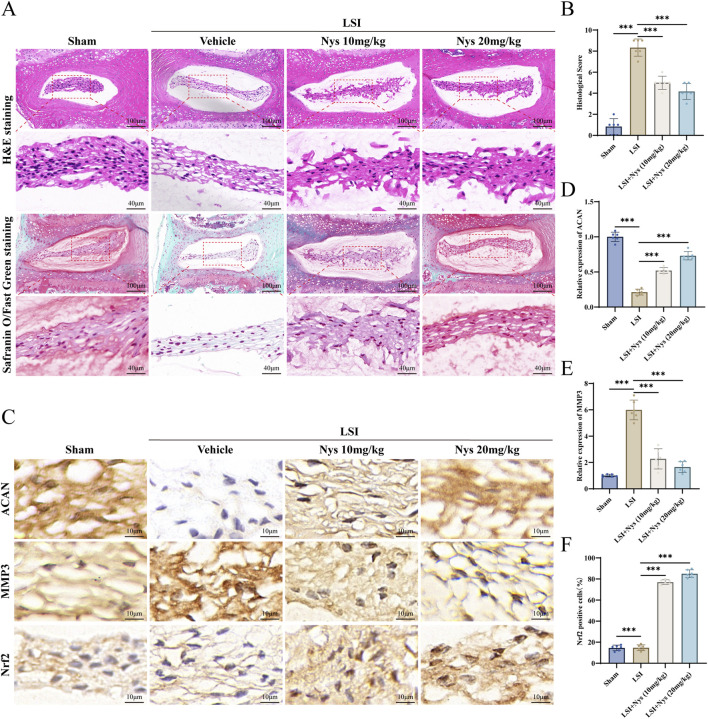
Nystose (Nys) alleviates nucleus pulposus (NP) degeneration and delays intervertebral disc degeneration (IDD) progression *in vivo*. **(A)** Haematoxylin and eosin (HE) staining and Safranin O-Fast green (SO) staining were performed 8 weeks after intraperitoneal injection (scale bar = 100 μm/40 μm; n = 6). **(B)** Histological scores of intervertebral discs in each group. **(C–F)** Representative immunohistochemical images and quantitative analysis of ACAN, MMP3, and Nrf2 expression in intervertebral disc tissues (scale bars = 10 μm). The average percentage of positively stained area was quantified using ImageJ software (n = 6). The data are expressed as the mean ± standard deviation. Statistical significance was determined using Student’s t-tests for two groups or one-way ANOVA for multiple comparisons. ***p < 0.001.

To verify the protective effect of Nys against IDD *in vivo*, we established a mouse model of IDD induced by LSI and administered Nys. First, the histological morphology of the intervertebral discs was assessed via HE staining. In the sham group, the disc structure was intact, with an oval-shaped NP rich in cells and a clear boundary with the AF. In the LSI model group, a significant reduction in the NP volume and shrinkage of the NP were observed, accompanied by a decrease in the number of NP cells, disordered arrangement in the AF, and a blurred NP-AF boundary. However, Nys significantly alleviated these changes, as indicated by a relatively large NP, increased cell numbers, a more intact AF structure, and relatively clear boundaries. These results indicate that Nys can effectively alleviate the structural destruction of the intervertebral disc induced by LSI. Moreover, the proteoglycan content was evaluated using SO staining. Compared with those in the Sham group, the degree of matrix degradation and proteoglycan loss in the LSI model group markedly decreased. Nys preserved stronger orange‒red staining, indicating that Nys effectively inhibited NP matrix degradation and maintained extracellular matrix homeostasis (Figures 7A,B). We subsequently used IHC staining to detect changes in the expression of the matrix synthesis marker ACAN, the matrix degradative enzyme MMP3, and the key transcription factor Nrf2 in NP tissue (Figures 7C–F). Compared with the sham group, the LSI model group showed a significantly weaker ACAN-positive signal and markedly more MMP3-positive staining, indicating the inhibition of matrix synthesis and increased matrix degradation. Compared with that in the LSI model group, ACAN-positive staining in the Nys intervention group significantly increased, whereas MMP3-positive staining significantly decreased. Furthermore, the expression of Nrf2 in the sham group was normal. In the LSI model group, no significant increase in Nrf2 staining was observed, suggesting that the antioxidant defence system may have been impaired during the degeneration process. In contrast, Nys markedly increased Nrf2-positive staining and induced significant nuclear translocation within the NP, indicating that Nys can activate the Nrf2 signalling pathway *in vivo*. In summary, Nys activates the Nrf2 signalling pathway to promote matrix synthesis and inhibit matrix degradation, thereby exerting a protective effect by delaying intervertebral disc degeneration *in vivo*.

## 4 Discussion

IDD is a degenerative disease that severely threatens the quality of life of middle-aged and elderly individuals (Francisco et al., 2022). Its global prevalence continues to increase (Hartvigsen et al., 2018), and it is characterized primarily by an imbalance in ECM homeostasis, NP cell dysfunction, and progressive structural destruction (Li et al., 2022). In this study, we predicted 15 overlapping targets between Nys and IDD through network pharmacology analysis. These targets were significantly enriched in signalling pathways such as the oxidative stress response, transcription factor binding, and metal ion regulation, suggesting that Nys participates in the pathogenesis of IDD through multiple pathways.

On this basis, we utilized TBHP to construct an oxidative stress model in NP cells. TBHP is a lipophilic organic peroxide that can easily cross the cell membrane to enter the cell interior directly, and the relatively unstable peroxy bond breaks intracellularly to generate free radicals; therefore, TBHP is widely used as an oxidative stress inducer (Ghosh et al., 2021; Xu et al., 2025). At the cellular level, Nys scavenges excess ROS whose production is induced by TBHP, increases GSH levels and antioxidant enzyme expression, and reduces MDA production. These antioxidant effects do not occur in isolation but appear to play a role in determining the functional fate of NP cells. Cells treated with Nys exhibited a recovery of ECM synthesis capacity and downregulation of catabolic enzyme expression. This phenomenon suggests that Nys maintains the balance of ECM synthesis and catabolism in NP cells by restoring a redox microenvironment conducive to cell survival.

The enrichment of the “metal ion regulation” pathway prompted us to further explore the regulatory effect of Nys on ferroptosis. As one of the forms of cell death most directly related to oxidative stress, ferroptosis has recently been confirmed as a key mechanism driving NP cell loss and IDD progression (Zhang Y. et al., 2022). Excessive ROS can impair the function of iron metabolism-related proteins, leading to iron overload, which in turn amplifies ROS generation through the Fenton reaction. Ultimately, this imbalance in iron homeostasis and oxidative stress results in a vicious cycle (Coradduzza et al., 2023; Sun et al., 2023; Plascencia-Villa and Perry, 2021). Our study revealed that Nys, on the one hand, helped scavenge ROS and, on the other hand, restored iron homeostasis by regulating TFR, FTL, and SLC40A1, thereby preventing the amplification effect of iron overload on oxidative damage.

These findings prompted us to investigate the core molecular mechanism through which Nys regulates iron homeostasis. As a core node predicted by network pharmacology analysis, Nrf2 critically regulates both the cellular antioxidant response and the expression of iron metabolism-related genes (Zhang X. et al., 2024; Li C. et al., 2024; Yu et al., 2026). Mechanistically, Nys promoted Nrf2 nuclear translocation, thereby upregulating its downstream targets HO-1 and GPX4. As a classic target, HO-1 generates catalytic products (biliverdin/bilirubin) that directly scavenge ROS (Ryter, 2022), whereas GPX4 restricts membrane lipid peroxides to dictate cellular sensitivity to ferroptosis (Liu et al., 2023). Crucially, silencing Nrf2 abrogated the ability of Nys to regulate iron metabolism-related proteins (SLC40A1, TFR, and FTL) and ECM homeostasis, suggesting that Nrf2 serves as a key hub connecting the antioxidant and antiferroptotic effects of Nys. Although the Nrf2/HO-1/GPX4 axis is a well-established therapeutic target for IDD (Li Z. et al., 2024), Nys offers distinct advantages. First, unlike lipophilic polyphenols, which have limited bioavailability, the polar oligosaccharide structure of Nys ensures its excellent water solubility and systemic absorption (Bali et al., 2015). Second, molecular docking and CETSA suggested that Nys binds to Nrf2 to facilitate its nuclear translocation. Finally, because it is derived from *Morinda officinalis* How., Nys has a long history of safe use in traditional medicine, making it ideal for the long-term management of IDD and overcoming the narrow therapeutic windows of synthetic agents (Pawlus and Kinghorn, 2007).

We further observed that endogenous Nrf2 in the NP tissue of mice with IDD induced by LSI might have been excessively consumed, resulting in the inability of the cells to effectively initiate protective transcriptional programs, accompanied by a reduction in the levels of ECM components and destruction of the disc structure. Although LSI induces degeneration primarily through mechanical instability, this abnormal biophysical loading rapidly triggers a cascade of secondary biochemical insults, particularly driving severe oxidative stress within the intervertebral disc microenvironment (Ling et al., 2025). Therefore, while it cannot directly correct anatomical mechanical defects, Nys restores Nrf2 nuclear translocation and reactivates downstream antioxidant and iron homeostasis regulatory networks, thereby restoring the balance of ECM metabolism at the tissue level and maintaining structural integrity.

Despite the various positive findings reported in this study, several limitations remain. First, the direct binding mode between Nys and Nrf2 has not yet been confirmed by more precise physicochemical methods, such as surface plasmon resonance or microscale thermophoresis, and the results of molecular docking simulations and CETSAs require further validation. Second, ferroptosis is a complex regulatory process involving multiple genes and levels. We focused primarily on the Nrf2/HO-1/GPX4 axis, but whether Nys also regulates ferroptosis through other pathways (such as the ferroptosis suppressor protein 1/coenzyme Q10 axis or the GTP cyclohydrolase 1/tetrahydrobiopterin axis) deserves in-depth exploration in the future (Jiang et al., 2021). Third, our *in vivo* evaluations were primarily based on histological and immunohistochemical analyses. In future studies, macroscopic imaging techniques (such as magnetic resonance imaging or micro-CT), biomechanical testing, and behavioural assessments for radicular pain should be incorporated to comprehensively evaluate functional recovery. Furthermore, the 8-week observation period provides only a midterm assessment, and the LSI model fails to fully replicate the complex, multifactorial aetiology of clinical IDD. Therefore, longer-term studies utilizing spontaneous or ageing-related degeneration models are necessary to further verify the translational relevance of these findings. Fourth, the *in vivo* pharmacokinetics and tissue bioavailability of Nys following systemic administration remain important topics. Although the healthy intervertebral disc is inherently avascular, LSI-induced degeneration frequently triggers endplate microfractures and neovascularization, which may locally increase the permeability of the disc to circulating drugs (Yan et al., 2025). Hence, prior to clinical translation, LC‒MS/MS studies to quantify the exact concentrations of Nys in both serum and disc tissues are crucial for definitively confirming the local bioavailability of Nys. Finally, although activating the Nrf2/HO-1/GPX4 axis robustly protects the intervertebral disc, Nrf2 acts as a biological “double-edged sword”. Prolonged systemic Nrf2 activation can inadvertently promote premalignant cell survival or chemoresistance (Wu et al., 2019). Because Nys was administered systemically to our *in vivo* model, the potential oncogenic and off-target effects of chronic Nrf2 hyperactivation require rigorous evaluation. Consequently, in future studies, the long-term systemic safety of Nys must be assessed, and localized delivery systems must be explored to restrict Nrf2 activation exclusively to the degenerative disc, maximizing efficacy while minimizing systemic risk.

**FIGURE 8:**
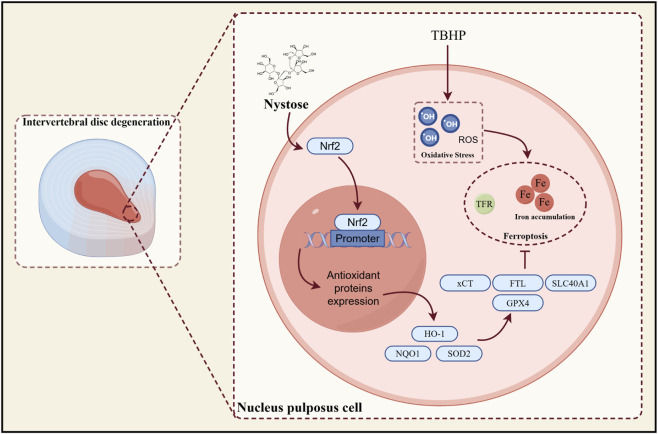
Schematic representation of the ability of nystose (Nys) to mitigate intervertebral disc degeneration. Nys inhibits ferroptosis and oxidative stress in nucleus pulposus cells by promoting the activation of Nrf2/HO-1/GPX4 signalling.

In summary, the results of this study suggest that Nys can inhibit oxidative stress and ferroptosis by activating the Nrf2/HO-1/GPX4 signalling pathway, thereby restoring the balance of ECM metabolism in NP cells and possibly delaying the progression of IDD in mice (Figure 8). These findings offer a potential theoretical basis and experimental foundation for the use of Nys as a novel and safe natural product-derived therapeutic agent for IDD intervention.

## 5 Conclusion

By integrating network pharmacology analysis with both *in vitro* and *in vivo* experiments, this study suggests the therapeutic potential of the natural small-molecule compound Nys for IDD. We observed that Nys can activate the Nrf2/HO-1/GPX4 signalling pathway, which inhibits both oxidative stress and ferroptosis. This mechanism restores redox homeostasis and iron metabolism balance in NP cells, thereby maintaining the equilibrium between ECM synthesis and catabolism. *In vivo* experiments further confirmed that Nys may delay the progression of IDD and preserve histological structural integrity through the activation of the Nrf2 signalling pathway. As a natural product with a clear origin and a favourable safety profile, Nys shows promise as a candidate drug for IDD treatment, providing a theoretical and experimental foundation for its future clinical translation.

## Data Availability

The datasets presented in this study can be found in online repositories. The names of the repository/repositories and accession number(s) can be found in the article/supplementary material.
